# Presbyopic correction on the cornea

**DOI:** 10.1186/s40662-014-0005-z

**Published:** 2014-11-13

**Authors:** Samuel Arba Mosquera, Jorge L Alió

**Affiliations:** SCHWIND eye-tech-solutions, Kleinostheim, Germany; Vissum Corporation, Alicante, Spain; Division of Ophthalmology, Universidad Miguel Hernández, Alicante, Spain

**Keywords:** Presbyopic refractive correction, Presbylasik, Corneal pseudoaccommodation, Corneal multifocality, Distance corrected near visual acuity, Monovision, Laser Blended Vision, Supracor and Intracor, KAMRA, PresbyMAX

## Abstract

**Purpose:**

The aim of this systematic review was to synthesize and appraise the evidence of the benefits of presbyopic correction on the cornea for visual function.

**Summary:**

Comprehensive search was conducted in MEDLINE using keywords like “presbylasik”, “presbyopic refractive surgery”, “corneal pseudoaccommodation” and “corneal multifocality”. We reviewed corrected and uncorrected visual acuities for distance and near (uncorrected distance visual acuity (UDVA), uncorrected near visual acuity (UNVA), corrected distance visual acuity (CDVA), distance corrected near visual acuity (DCNVA), corrected near visual acuity (CNVA)), along with the refractive outcomes in spherical equivalent (SE) and astigmatism comparing the differences observed between preoperative myopic and hyperopic patients, as well as among techniques.

Thirty-one studies met the inclusion and quality criteria. Monovision provides excellent distance and near uncorrected acuities, but with a 17% retreatment and a 5% reversal rate. Initial multifocal ablations result in 12% loss of 2 or more lines of CDVA, and a 21% retreatment rate. Laser Blended Vision provides excellent UDVA, but with a 19% retreatment rate. Initial experiences with Supracor show moderate predictability and a 22% retreatment rate. Intracor results in 9% loss of 2 or more lines of CDVA. KAMRA provides excellent UDVA, with only a 1% retreatment rate, but a 6% reversal rate. Initial experiences with PresbyMAX provided excellent UNVA and DCNVA, showing excellent predictability and a 1% reversal rate.

**Conclusions:**

The findings have implications for clinicians and policymakers in the health-care industry and emphasize the need for additional trials examining this important and widely performed clinical procedure.

## Introduction

Surgical presbyopic correction has seen a tremendous increase in interest in the recent times. The application of excimer laser systems in surgically correcting presbyopia is as old as the laser refractive surgery [1]. Refractive surgeons have faced challenges in effectively combining the treatment of refractive errors and presbyopia.

Moreira et al. said in 1993 [1]: “After multifocal ablations, a greater spread of surface powers is observed, often with a bimodal distribution, indicative of an apparent multifocal effect. These observations suggest that in some patients undergoing photorefractive keratectomy (PRK) for myopia, it may be possible to reduce symptoms of presbyopia”.

The aim of this systematic review was to synthesize and appraise the evidence of benefits of presbyopic correction on the cornea for visual function.

### History of development

We categorized the found techniques as:

#### Monovision

Monovision is another extended technique [1] where the dominant eye is corrected for distance usually. Alternatively, crossed monovision [2] involves correcting the dominant eye for near vision and offers the patients better near vision with minimal compromise in stereo acuity and overall high patient satisfaction.

#### Multifocal ablations

Vinciguerra et al. [3] proposed a 10 to 17 micron deep semilunar-shaped zone immediately below the pupillary center, steepening the corneal curvature in that area and reported promising results with this technique [4].

A pseudo-accommodative cornea is realized basically in the form of a peripheral near zone (concentric ring for near vision) [5] or in the form of a central near zone (central disc for near vision) [6]. This modified form of correction introduced advancements in the prebyopic treatments.

Charman [7] concluded that the main requirement in presbyopia treatments is extended binocular depth-of-focus to yield adequate distance and near vision with good retinal contrast at lower spatial frequencies. He further suggested that, for many presbyopes, this can be achieved by aiming for residual higher-order aberrations (HOAs). The highest levels of acuity and modulation transfer function at a single distance, usually targeted in other kinds of refractive procedures are not prioritized in presbyopia corrections.

Artola et al. [8] found evidence for delayed presbyopia caused by induced corneal aberrations after PRK for myopia. These aberrations might reduce the quality of the retinal image for distance, but enhance the near acuity through multifocal effect. This can delay the onset of age-related near vision symptoms.

Dai [9] was one of the first to propose the use of rigorous methodologies to theoretically optimize vision over the entire target range from near to distance.

Ortiz et al. [10] characterized the optical quality by the Strehl ratio, the spot size on the retina, and objective decimal visual acuity calculated based on measured corneal topography using Fresnel propagation algorithm based on a realistic eye model. They found that in patients, who underwent central presbyLASIK treatment, it was possible to evaluate the optical quality through a complete characterization of the eye and a propagation algorithm (that takes into account all refractive surfaces in the eye at the same time) applied to this eye model.

PresbyLASIK is another important addition to the techniques of multifocal ablations. The term presbyLASIK indicates a corneal surgical procedure based on traditional laser in situ keratomileusis (LASIK) to create a multifocal surface able to correct any visual defect for distance while simultaneously reducing the near spectacle dependency in presbyopic patients [11,12].

Little literature is found concerning monocular distance corrected performance after presbyLASIK (i.e., best achievable distance vision combined with the pseudoaccommodation contribution for near).

In a previous report [13] , presbyLASIK was stated as a promising technology, but lacking the level of maturity of monovision. A combination of micro–monovision and presbyLASIK (i.e., both eyes multifocally corrected but with a focus shift between eyes, with the distance eye closer to emmetropia and the near eye more in myopia) has the potential to provide better intermediate vision. A higher stereoacuity can be expected from the multifocal ablations along with a lower compromise in uncorrected distance visual acuity (UDVA) due to the combination of monovision and blended vision.

In another report [14], statistically significant differences were observed between myopic and hyperopic cases after treating with bi-aspheric ablation profiles. The outcomes for the myopic cases were better in terms of postoperative spherical equivalent (SE) (−0.19 D more residual myopia for preoperative myopes), binocular uncorrected near visual acuity (UNVA) and distance corrected near visual acuity (DCNVA) (both 0.2 lines better for preoperative myopes), and change in corrected distance visual acuity (CDVA) (1 letter better for preoperative myopes).

#### Intracorneal multifocality

Intracorneal ablations in the form of concentric rings are used to produce a weaker region in the central part of the cornea resulting in a hyperpolate shape. This flapless procedure is restricted within the boundaries of the corneal stroma.

#### Corneal inlays

Three types of corneal inlays are available today for presbyopic treatments. The KAMRA (AcuFocus, Irvine, Calif.) corneal inlay uses a pinhole effect. PresbyLens (ReVision Optics, Lake Forest, Calif.) is based on microscopically changing the shape of the eye’s surface. Flexivue Microlens (Presbia, Los Angeles) is a refracting inlay with a refractive index different from the cornea.

Based on reports and presentations, near vision can be improved while retaining distance visual acuity with the KAMRA presbyopic inlay. Although corneal inlays are placed only in one eye, they differ from monovision by not compromising on the distance vision.

However, inlays also represent a compromise. For the KAMRA, light entering the eye is restricted, which may reduce contrast and night vision, and there can be optical side effects. The benefit of using inlays is the ability to remove and reverse the effects of the treatment. Intracorneal inlay and simultaneous refractive surgery has also presented safe and efficacious results in patients with presbyopia and emmetropia.

#### Hybrid techniques

Hybrid techniques are designed to combine the benefits of two of the above-mentioned approaches and suppress the related disadvantages. These hybrid modifications include: conductive keratoplasty (CK) (full correction in distance eye combined with CK multifocality and monovision in the near eye), Supracor and PresbyMAX (reduced multifocality in distance eye combined with full multifocality and monovision in the near eye), Intracor (full correction in distance eye combined with Intracor multifocality and monovision in the near eye), KAMRA (full correction in distance eye combined with pinhole based extended depth-of-focus and monovision in the near eye), as well as laser blended vision (moderate multifocality in both eyes combined with monovision in the near eye).

Reinstein et al. [15] successfully combined extended depth of focus with monovision in a micro-monovision protocol, whereas Epstein and Gurgos [16] combined monocular peripheral presbyLASIK on the non-dominant eye with monofocal distance correction on the dominant eye.

## Review

We conducted a comprehensive search in MEDLINE using keywords like “presbylasik”, “presbyopic refractive surgery”, “corneal pseudoaccommodation” and “corneal multifocality”.

We reviewed corrected and uncorrected visual acuities for distance and near (UDVA, UNVA, CDVA, DCNVA, corrected near visual acuity (CNVA)), along with the refractive outcomes in SE and astigmatism comparing the differences observed between preoperative myopic and hyperopic patients, as well as among techniques. We used the standard equivalent Snellen acuities for distance vision (20/x, where 20/20 represents 0.0 logMAR) combined with the revised Jaeger scale for near vision (Jn, where J1 represents 0.0 logMAR and 0.0 logRAD).

Thirty-one studies met the inclusion and quality criteria.

### Monovision

This is probably the most extensively used and old (but not outdated) technique for alleviating presbyopic symptoms.

Ayoubi et al. [17] compared the visual outcomes, complications, and patient satisfaction after femtosecond LASIK and CK in a retrospective consecutive single-surgeon comparative study in private laser clinics in the United Kingdom and found that there were 3% and 50% retreatment rates after femtosecond LASIK and CK (P < .0001) respectively. On a questionnaire administered at 12 months, 20 patients (62.5%) in the femtosecond LASIK group and 11 patients (34.4%) in the CK group reported being satisfied (P = .02). They concluded that in emmetropic presbyopic cases, femtosecond LASIK monovision provided stable correction with less induced astigmatism and HOAs. Eyes with CK monovision had regression and induced astigmatism.

Braun, Lee and Steinert [2] evaluated the preoperative characteristics and postoperative outcomes of prepresbyopic patients selecting monovision correction by LASIK in a retrospective observational case series of 172 sequentially treated myopic and hyperopic patients, 45 years or older, who sought LASIK vision correction with the goal of attaining monovision. They found out that LASIK monovision correction represents a viable and increasingly popular method of correcting presbyopic and prepresbyopic patients considering refractive surgery. Crossed monovision may be applied successfully to appropriately chosen patients. The distance vision eye in the monovision patient may have a lower tolerance for residual refractive error and require a higher rate of enhancements than a standard laser vision correction patient.

Reilly et al. [18] assessed the success of surgical monovision in presbyopic patients in a retrospective chart review of 82 patients who elected to undergo surgical monovision with LASIK conducted between January 2000 and January 2003. Postoperative SE in the distance eyes was −0.01 (standard deviation (SD), 0.38) and in the near eyes −1.24 (SD, 0.91). There were 6 enhancements in the near eyes (7%) and 17 enhancements in the distance vision eyes (21%). This difference was statistically significant (P = 0.007). Thirty patients underwent a contact lens trial of monovision before LASIK, and none of those patients elected monovision reversal. There were 52 patients who did not undergo a contact lens monovision trial before LASIK monovision, and 2 of these patients underwent monovision reversal. Monovision success in this population was 97.6%.

Wright et al. [1] measured binocular function and patient satisfaction with monovision induced by PRK in 21 myopic presbyopic patients. In the monovision group at near and distance, 20 patients (95.3%) had binocular visual acuity of 20/25 or better. No patient in the monovision group used reading glasses postoperatively. All patients maintained binocular fusion and stereo acuity ranging from 40 to 800 seconds of arc. Mean patient satisfaction was 86% (range 40% to 100%).

See Table 1 for a comparison of monovision reported outcomes.

**Table 1:** **Summary of clinical studies in monovision**

**Study**	**n**	**Follow-up**	**UDVA**	**UNVA**	**Retreatment**	**Reversal**
Goldberg [19]	114	>6M	76% > 20/25	96% > J2	13%	2%
Goldberg [20]	137	>6M	89% > 20/25	99% > J2	15%	6%
Braun et al. [2]	172	---	---	---	28%	7%
Garcia-Gonzalez M. et al. [21]	37	---	20/19	J1	---	---
Ito M et al. [22]	54	5Y	20/20	J1	---	---
			98% > 20/25	76% > J2		
**Grand total**	**514**	**6M-5Y**	**20/20**	**J1**	**17%**	**5%**
			**87% > 20/25**	**90% > J2**		

UDVA: uncorrected distance visual acuity, UNVA: uncorrected near visual acuity, n: number of patients in the study, M: months, Y: years, J: Jaeger scale for near vision.

### Conductive keratoplasty

Stahl [23] assessed the long-term safety, efficacy, and stability of CK in the unilateral treatment of presbyopia performed in the non-dominant eye of ten near-plano presbyopic patients (6 women and 4 men) to improve their near vision. Three years after CK, the mean near uncorrected visual acuity (UCVA) was J3. The mean manifest refraction spherical equivalent (MRSE) at 3 years was −1.06 ± 0.81 dioptres(D), which represents a 0.25 D change from the MRSE at 1 year. They reported a +0.26 D change in MRSE in the dominant untreated eyes during a period of 3 years. This change is not statistically different compared to the CK-treated eyes during the 3-year postoperative period. Seventy-eight percent eyes reported binocular distance UCVA 20/20 or better and near UCVA J3 or better. In their cohort, no eye lost best spectacle-corrected visual acuity (BSCVA) or had induced cylinder greater than or equal to 0.75 D. They also reported a stable keratometry with 45.09 D compared to 45.08 D reported 3 and 1 year post operatively.

Ye et al. [24] evaluated the visual outcomes of CK for relief of symptomatic presbyopia of pseudophakia with monofocal intraocular lens implantation on 27 eyes of 27 patients. Twelve months after CK, the binocular UNVA was significantly improved from logMAR 0.88 ± 0.16 preoperatively to logMAR 0.30 ± 0.13 (P < .05); the binocular UDVA and BSCVA remained unchanged; MRSE was significantly reduced from 0.01 ± 0.68 D preoperatively to −1.68 ± 0.39 D (P < .05).

### Multifocal ablations

#### Initial

##### Central myopia

Jackson, Tuan, and Mintsioulis [25] evaluated an aspheric ablation profile to improve near vision in presbyopic patients with hyperopia in a prospective, nonrandomized, clinical trial of 66 eyes of 33 hyperopic patients who underwent customized bilateral refractive surgery, which included an aspheric presbyopia treatment shape and wavefront-driven hyperopic treatment. Sixty eyes completed 6-month and 50 eyes completed 12-month postoperative follow-up. At 6 months, mean CDVA was 20/20 ± 1 line (range: 20/25 to 20/10). Mean gain in DCNVA was 2.7 ± 1.7 lines with a maximum of 6 lines of near. Spectacle dependence for tasks, such as reading and computer use, was reduced. At 12 months, 100% of patients had achieved binocular simultaneous uncorrected vision of 20/25 or better and J3. Refraction was stable over 12 months. Contrast sensitivity reduction was clinically insignificant (1 step or 0.15 logCS). Negative spherical aberration highly correlated with postoperative improvement of DCNVA. Patients who had a larger amount of preoperative hyperopia or a greater decrease of preoperative DCNVA were more likely to have overall satisfaction.

##### Peripheral myopia

Pinelli et al. [26] analyzed the results of a peripheral presbyLASIK algorithm for the correction of presbyopia in 22 hyperopic patients. Six months postoperatively, mean binocular UCVA was 1.06 ± 0.13 for distance and 0.84 ± 0.14 for near. Mean postoperative SE refraction was −0.42 D (range: −1.12 to +0.87 D). Two (4.5%) eyes lost 1 line of BSCVA for distance and near vision, and 20 (45%) eyes gained 1 line of distance BSCVA. Contrast sensitivity decreased for 3, 6, 12, and 18 cycles/degree. Corneal aberration analysis showed a slight increase in coma and decrease in spherical aberration.

Uy and Go [27] investigated the refractive outcomes and spherical aberration of multifocal LASIK to create a distant-dominant center and near-dominant periphery in 195 eyes with myopic presbyopia and 119 eyes with hyperopic or emmetropic presbyopia that underwent LASIK or epi-LASIK. The mean postoperative SE refraction was −0.40 ± 0.77 D for myopic presbyopia and +0.15 ± 0.62 D for hyperopic or emmetropic presbyopia. They defined functional vision as 20/30 or better UDVA combined with J3 or better near UCVA. They achieved functional vision in 162 (83%) eyes with myopic presbyopia and 103 (87%) eyes with hyperopic or emmetropic presbyopia. They observed 0.312 microns of induced spherical aberration (at 6.0 mm optical zone) for myopic presbyopia treatments and 0.016 microns for hyperopic presbyopia treatments.

See Table 2 for a comparison of multifocal reported outcomes.

**Table 2:** **Summary of clinical studies in multifocal ablations**

**Study**	**n**	**Follow-up**	**UDVA**	**UNVA**	**DCNVA**	**CDVA**	**CNVA**	**Refr.Outc.**	**Retreat.**
Vinciguerra et al. [3]	3	2Y	---	J3 100%	---	---	---	---	---
Alió et al. [10]	50	6M	20/20	J4	J5	14% < −2lns	14% < −2lns	64% ± 0.5DS	12%
			88% > 20/25	92% > J3	28% > J3				
Jung SW et al. [28]	28	6M	93% > 20/25	64% > J3	---	---	---	---	---
Epstein and Gurgos [16]	103	1Y	74% > 20/25	74% > J2	---	---	---	---	28%
Jackson et al. [25]	50	1Y	100% > 20/25	86% > J2	70% > +2lns	10% < −2lns	8% < −2lns	88% ± 0.5DS	---
**Grand total**	**234**	**6M-2Y**	**20/20**	**J4**	**J5**	**12% < −2lns**	**11% < −2lns**	**76% ± 0.5DS**	**21%**
			**87% > 20/25**	**81% > J3**	**49% > +2lns**				

UDVA: uncorrected distance visual acuity, UNVA: uncorrected near visual acuity, n: number of patients in the study, DCNVA: distance-corrected near visual acuity, CDVA: corrected distance visual acuity, CNVA: corrected near visual acuity, Refr.Outc.: refractive outcome, Retreat.: retreatment rate, M: months, Y: year(s), J: Jaeger scale for near vision, lns: Snellen lines, DS: dioptre sphere.

#### Supracor

Ryan and O’Keefe [29] reported the safety and efficacy of treatment of hyperopic presbyopia with Supracor on 46 eyes (23 patients). They reported a mean binocular UDVA of 0.07 logMAR ±0.12 (SD) at 6 months, with 91% eyes achieving a binocular UDVA of 0.2 logMAR or better. In terms of reading ability, 91% eyes reported an uncorrected reading ability of N8 or better. Postoperatively, 93% eyes required no reading glasses. In terms of CDVA, 6% eyes lost 2 or more lines while 100% eyes maintained a CDVA of 0.2 logMAR or better. In their cohort, 22% eyes required a retreatment in the dominant eye to enhance UDVA. They reported that 96% patients were happy that they had the procedure indicating patient satisfaction.

See Table 3 for a comparison of Supracor reported outcomes.

**Table 3:** **Summary of clinical studies in Supracor**

**Study**	**n**	**Follow-up**	**UDVA**	**UNVA**	**DCNVA**	**CDVA**	**CNVA**	**Refr.Outc.**	**Retreat.**
O’Keefe et al. [29]	46	6M	20/23	J3	---	7% < −2lns	---	54% ± 0.5DS	13%
			78% > 20/25	91% > J3				46% ± 0.5DC	
Cosar CB et al. [30]	123	6M	20/23	J2	---	11% < −2lns	0% < −2lns	---	---
			37% > 20/25	89% > J2					
**Grand total**	**169**	**6M**	**20/23**	**J3**	**---**	**9% < −2lns**	**0% < −2lns**	**54% ± 0.5DS**	**13%**
			**58% > 20/25**	**90% > J3**				**46% ± 0.5DC**	

UDVA: uncorrected distance visual acuity, UNVA: uncorrected near visual acuity, n: number of patients in the study, DCNVA: distance-corrected near visual acuity, CDVA: corrected distance visual acuity, CNVA: corrected near visual acuity, Refr. Outc.: refractive outcome, Retreat.: retreatment rate, M: months, J: Jaeger scale for near vision, lns: Snellen lines, DS: dioptre sphere, DC: dioptre cylinder.

#### PresbyMAX

Uthoff et al. [31] investigated the outcomes of simultaneous correction of presbyopia and ametropia by a bi-aspheric cornea modulation technique. Their treatment technique was based on the creation of a central hyperpositive area for near vision and leaving the pericentral cornea for far vision. They reported the outcomes in 60 eyes of 30 patients treated with the PresbyMAX technique. They observed an improvement in the mean binocular distance of uncorrected visual acuity (DUCVA) in the hyperopic group from 0.28 ± 0.29 logMAR to −0.04 ± 0.07 logMAR, in the emmetropic group from −0.05 ± 0.07 logMAR to 0.02 ± 0.11 logMAR, and in the myopic group from 0.78 ± 0.27 logMAR to 0.09 ± 0.08 logMAR. They also reported an increment in the mean binocular near uncorrected visual acuity (NUCVA) in the hyperopic group from 0.86 ± 0.62 logRAD to 0.24 ± 0.23 logRAD, and in the emmetropic group from 0.48 ± 0.14 logRAD to 0.18 ± 0.11 logRAD. The myopic presbyopes however, showed a decrease of the mean binocular NUCVA from 0.04 ± 0.19 logRAD to 0.12 ± 0.18 logRAD. Postoperatively, they reported a mean SE for distance refraction of −0.13 ± 0.61 D for the hyperopic presbyopia, −0.43 ± 0.35 D for the emmetropic presbyopia, and −0.68 ± 0.42 D for the myopic presbyopia group. The treatment planning was aimed at −0.50 D in all groups. However, they also reported up to 13% eyes losing 2 lines of CDVA.

See Table 4 for a comparison of PresbyMAX reported outcomes.

**Table 4:** **Summary of clinical studies in PresbyMAX**

**Study**	**n**	**Follow-Up**	**UDVA**	**UNVA**	**DCNVA**	**CDVA**	**CNVA**	**Refr.Outc.**	**Retreat.**	**Revers.**
Uthoff et al. [31]	60	6M	20/21	J3	---	13% < −2lns	0% < −2lns	73% ± 0.5DS	0%	0%
			83% > 20/25	43% > J2						
Luger et al. [13]	66	1Y	20/23	J1	J3	3% < −2l ns	8% < −2 lns	73% ± 0.5DS	0%	0%
			70% > 20/25	84% > J2				93% ± 0.5DC		
Baudu [14]	716	6M	20/25	J1	J2	6% < −2l ns	1% < −2 lns	88% ± 0.5DS	14%	<1%
			76% > 20/25	90% > J2				99% ± 0.5DC		
**Grand total**	**842**	**6M-1Y**	**20/24**	**J1**	**J3**	**5% < −2lns**	**3% < −2lns**	**85% ± 0.5DS**	**10%**	**1%**
			**77% > 20/25**	**90% > J2**	**38% > +2lns**			**98% ± 0.5DC**		

UDVA: uncorrected distance visual acuity, UNVA: uncorrected near visual acuity, n: number of patients in the study, DCNVA: distance-corrected near visual acuity, CDVA: corrected distance visual acuity; CNVA: corrected near visual acuity, Refr. Outc.: refractive outcome; Retreat.: retreatment rate, Revers.: percentage of reverse treatment, M: months, Y: year, J: Jaeger scale for near vision, lns: Snellen lines, DS: dioptre sphere, DC: dioptre cylinder.

### Intracorneal multifocality

Bohac et al. [32] reported early outcomes of a cohort of presbyopic patients treated with Intracor in 95 eyes (49 patients with non-dominant eye and 23 with bilateral treatment). At 3 months of postoperative follow-up, all patients gained several lines of UNVA with monocular UNVA Jaeger system 1.67 ± 0.28. UDVA showed slight improvement over time and initial myopic shift showed tendency of slight decrease with all patients achieving 1.0. Overall, patient satisfaction was very high (98%) with only a few (3 patients, 5 eyes) reporting mild halo and glare at 3 months postoperative.

Holzer et al. [33] investigated early functional outcomes of the Intracor femtosecond laser-based intrastromal procedure to treat presbyopia on 25 eyes of 25 presbyopic patients. They reported uneventful surgeries and postoperative healing, an increase in the mean postoperative UNVA from 0.7 ± 0.16 logMAR to 0.26 ± 0.21 logMAR and a marginal change in the mean postoperative UDVA from 0.11 ± 0.11 logMAR to 0.05 ± 0.1 logMAR at 3 months. In their cohort, the mean sphere changed from +0.75 ± 0.23 D preoperatively to +0.15 ± 0.31 D postoperatively and mean cylinder from −0.33 ± 0.17 D to −0.42 ± 0.23 D. In all the eyes, they observed that the cornea was clear within a few hours after surgery without any remaining cavitation gas bubbles.

See Table 5 for a comparison of Intracorneal multifocality reported outcomes.

**Table 5:** **Summary of clinical studies in Intracor**

**Study**	**n**	**Follow-Up**	**UDVA**	**UNVA**	**DCNVA**	**CDVA**	**CNVA**
Ruiz LA et al. [34]	83	6M	20/20 89% > 20/25	J2 100% > J2	J2	2% < −2lns	---
Holzer et al. [33]	25	3M	20/22 83% > 20/25	J4 43% > J2	J3	8% < −2lns	---
Mike P. Holzer et al. [35]	58	1Y	20/25 61% > 20/25	J3 40% > J2	---	7% < −2lns	11% < −2lns
Menassa N et al. [36]	23	18M	20/32	J3	---	26% < −2lns	---
**Grand total**	**189**	**6M-18M**	**20/24 77% > 20/25**	**J3 66% > J2**	**J2**	**9% < −2lns**	**11% < −2lns**

UDVA: uncorrected distance visual acuity, UNVA: uncorrected near visual acuity; n: number of patients in the study, DCNVA: distance-corrected near visual acuity, CDVA: corrected distance visual acuity, CNVA: corrected near visual acuity, M: months, Y: year, J: Jaeger scale for near vision, lns: Snellen lines.

### Corneal inlays

Lindstrom et al. [37] provided an overview of the three types of corneal inlays currently used for the correction of presbyopia. They reviewed recently published evidence on the safety and efficacy of corneal inlays. They found that the results for corneal reshaping and refractive inlays are promising although very limited. Small-aperture inlays are often used today owing to the positive outcomes in terms of improved uncorrected near and intermediate vision without a significant loss in distance acuity or an unacceptable increase in visual symptoms. Their reviewed literature suggested minimal complications using inlays, and the ability to remove the inlays if necessary, being an important feature. Inlays do not prevent visualization or imaging of the retina and may be retained during subsequent cataract surgery. They concluded that there is currently no other effective solution for the large and increasing demographic of presbyopes who desire good uncorrected vision at all distances without the risks of intraocular surgery or the visual compromises of monovision. Based on current developments, corneal inlays can be considered to have a bright future.

Tomita et al. [38] evaluated the visual outcomes after implantation of a Kamra small-aperture corneal inlay into a femtosecond-created corneal pocket to treat presbyopia in patients who had previous LASIK (223 eyes from 223 patients). The mean UDVA in the operated eye decreased 1 line from 20/16 preoperatively to 20/20 6 months postoperatively (P < .001). The mean UNVA improved 4 lines from J8 to J2 (P < .001). At 6 months, significant improvements were observed in patient’s dependence on reading glasses and patient satisfaction with vision without reading glasses.

Yılmaz et al. [39] evaluated the long-term visual results of Acufocus ACI-7000 (now KAMRA) intracorneal inlay implantation in 39 presbyopic phakic patients. At the 4-year follow-up, all patients (N = 22) had 2 or more lines of improvement in UNVA with no significant loss in distance vision. They reported a mean final UNVA of 20/20 (Jaeger [J1]); with 96% of patients with reading ability of J3 or better. The uncorrected distance acuity was 20/40 or better in all eyes. In their cohort, five patients were reported with a progressing cataract and 2 patients presented no refractive change after the inlay implantation. Although they reported no complications during cataract extraction, 4 inlays were explanted during their study. Their study revealed no severe corneal complications affecting the eventual outcomes of the procedure.

Seyeddain et al. [40] evaluated the safety and efficacy of the AcuFocus Corneal Inlay 7000 (ACI 7000) implanted in emmetropic presbyopic patients for the improvement of near and intermediate vision. They reported the outcomes in 32 naturally emmetropic presbyopic patients with over 2-years of follow-up. After mean follow-up of 24.2 ± 0.8 months (range: 24 to 26 months), 96.9% of patients read J3 or better in the implanted eye. They observed an improvement in the mean binocular UNVA from J6 preoperatively to J1 after 24 months. Mean binocular uncorrected intermediate visual acuity (UIVA) was 20/20 at 1 month and remained 20/20 throughout the 24-month follow-up. In their cohort, 71.9% eyes reached a UIVA of 20/20 or better. They observed a mean UDVA of 20/20 in the implanted eye and 20/16 binocularly, at 24 month follow-up. No inlay was explanted during the study. They reported recentration in 2 decentered inlays after 6 months of implantation. An insufficient increase in near and intermediate visual acuity indicated the need for recentration. These metrics showed significant improvements after recentration, for both the cases.

See Table 6 for a comparison of KAMRA reported outcomes.

**Table 6:** **Summary of clinical studies in KAMRA**

**Study**	**n**	**Follow-Up**	**UDVA**	**UNVA**	**DCNVA**	**CDVA**	**CNVA**	**Retreat.**	**Reversal**
Dexl AK et al. [41]	32	2Y	20/16	J3	---	---	---	---	---
Yılmaz et al. [39]	22	4Y	96% > 20/25	96% > J2	---	5% < −2lns	---	---	18%
Dexl AK et al. [42]	24	1Y	20/16 100% > 20/25	J2 92% > J3	---	4% < −2lns	8% < −2lns	0%	0%
Tomita M et al. [43]	64	6M	20/19 86% > 20/25	J2 60% > J2	---	5% < −2lns	2% < −2lns	1%	---
Dexl et al. [44]	24	2Y	20/16	J4	---	4% < −2lns	---	0%	0%
**Grand total**	**166**	**6M-4Y**	**20/17 93% > 20/25**	**J3 73% > J2**	**---**	**5% < −2lns**	**5% < −2lns**	**1%**	**6%**

UDVA: uncorrected distance visual acuity, UNVA: uncorrected near visual acuity, n: number of patients in the study, DCNVA: distance-corrected near visual acuity, CDVA: corrected distance visual acuity, CNVA: corrected near visual acuity, Retreat.: retreatment rate, M: months, Y: year(s), J: Jaeger scale for near vision, lns: Snellen lines.

### Hybrid techniques

Alarcon et al. [45] evaluated visual quality after LASIK performed to achieve monovision in presbyopic patients further targeting a postsurgical corneal asphericity of −0.80 in the dominant eye and −1.00 in the non-dominant eye. The study enrolled 25 patients (50 eyes) with a mean age of 49.3 years ±4.5 (SD). Postoperatively, more than 90% of patients had a binocular uncorrected distance and near visual acuity of 0.0 logMAR or better, although the contrast sensitivity function diminished, especially in the nondominant eye and with binocular vision. A significant decline was seen in the stereoacuity in all the patients (P < .001). The visual discrimination capacity under binocular conditions, declined in the non-dominant eyes (P < .005) but did not change significantly in the dominant eyes (P = .614). A non-significant increase (P > 0.5) was observed in the mean objective scatter index value postoperatively.

See Table 7 for a comparison of Laser Blended Vision reported outcomes.

**Table 7:** **Summary of clinical studies in laser blended vision**

**Study**	**n**	**Follow-up**	**UDVA**	**UNVA**	**DCNVA**	**CDVA**	**CNVA**	**Refr.Outc.**	**Retreat.**
Reinstein et al. [46] Myopes	272	1Y	20/17	J3	---	0% < −2lns	---	92% ± 0.5DS	19%
			99% > 20/25	96% > J3				92% ± 0.5DC	
Reinstein et al. [46] Hyperopes	222	1Y	20/18	J4	---	0% < −2lns	---	79% ± 0.5DS	22%
			99% > 20/25	81% > J3				82% ± 0.5DC	
Reinstein et al. [46] Emmetropes	176	1Y	20/17	J3	---	0% < −2lns	---	92% ± 0.5DS	16%
			99% > 20/25	95% > J3				85% ± 0.5DC	
**Grand total**	**670**	**1Y**	**20/17**	**J3**	**---**	**0% < −2lns**	**---**	**88% ± 0.5DS**	**19%**
			**99% > 20/25**	**91% > J3**				**86% ± 0.5DC**	

UDVA: uncorrected distance visual acuity, UNVA: uncorrected near visual acuity, n: number of patients in the study, DCNVA: distance-corrected near visual acuity, CDVA: corrected distance visual acuity, CNVA: corrected near visual acuity, Refr. Outc.: refractive outcome, Retreat.: retreatment rate, Y: year, J: Jaeger scale for near vision, lns: Snellen lines, DS: dioptre sphere, DC: dioptre cylinder.

## Conclusions

Monovision is highly rated by patients even though binocular vision is compromised [47].

Although the depth of focus acts as a useful marker, acuity at typical near vision distances is a more suitable metric that is closely related to patients’ real expectations and concerns [48]. High levels of patient satisfaction have been reported for monovision LASIK by Goldberg [19] (reporting 96% satisfaction) and Miranda [49] (reporting 92% satisfaction).

Contact lens monovision and LASIK-induced monovision traditionally use a nomogram for near addition, with the degree of anisometropia increasing from approximately −1.50 D for a 45-year-old patient up to −2.50 D for a 65-year-old patient [20].

CK seems to produce functional corneal multifocality with definable introduction of surgically induced astigmatism and higher-order optical aberrations, and development of a more prolate corneal contour. These optical factors may militate toward improved near vision function [50].

PresbyLASIK treatment constitutes a new modality in the correction of presbyopia after monovision LASIK [51,52]. In several reports [53,54], Alió et al. demonstrated the efficiency, predictability, stability, safety, and visual quality of central presbyLASIK in presbyopic patients with hyperopia.

Pinelli et al. [26] investigated the outcome of the correction of presbyopic patients with hyperopia using a peripheral presbyLASIK algorithm called Peripheral Multifocal LASIK (PML). This treatment creates a multifocal corneal profile in a 6.5 mm diameter zone by the combination of a positive ablation performed over a 6.5 mm zone and a negative ablation performed over an optical zone no smaller than 5.0 mm. The hypothesis is that the ring between the 5.0 and 6.5 mm optical zones provides multifocality.

Semoun et al. were the first to describe a P. acnes (Propionibacterium acnes) infection after a presbyopic LASIK procedure. This unusual case of infectious keratitis emphasizes the fact that even though such cases may rarely present in the clinics, the patients must be informed about the potential risks of such infections [55].

The reversibility of the presbyLASIK procedures have been viewed controversially. These viewpoints have been discussed in the work of Luger et al. [13]. In another study, Luger et al. [56] demonstrated the use of a nonwavefront-guided Presby reversal treatment targeting a monofocal cornea after bi-aspheric ablation profile in a patient intolerant to multifocality. Baudu et al. [14] have also reported good results in retreatments involving reversals after presbyLASIK.

Artola et al. found evidence for delayed presbyopia after PRK in a non-presbyLASIK protocol for myopia [8].

The initial results of Intracor raised the expectations for this technique. However, subsequent reports on corneal ectasia and concerns regarding the retreatment and reversal possibilities raised questions about the safety of this technique [57].

As for the corneal inlays, some more clinical and theoretical work has been published so far, including reading performance and patient satisfaction[44], optimum centration and residual defocus [58], binocular visual simulation [59], and vignetting and field of view with the KAMRA corneal inlay [60].

See Table 8 for a comparison across techniques.

**Table 8:** **Comparison across surgical techniques of presbyopic correction**

**Presbyopic approach**	**n**	**Follow-Up**	**UDVA**	**UNVA**	**DCNVA**	**CDVA**	**CNVA**	**Refr.Outc.**	**Retreat.**	**Reversal**
Monovision	514	6M-5Y	20/20	J1	---	---	---	---	17%	5%
			87% > 20/25	90% > J2						
Multifocal	234	6M-2Y	20/20	J4	J5	12% < −2lns	11% < −2lns	76% ± 0.5DS	21%	---
			87% > 20/25	81% > J3	49% > +2lns					
LBV	670	1Y	20/17	J3	---	0% < −2lns	---	88% ± 0.5DS	19%	---
			99% > 20/25	91% > J3				86% ± 0.5DC		
Supracor	169	6M	20/23	J2-J3	---	9% < −2lns	0% < −2lns	54% ± 0.5DS	13%	---
			58% > 20/25	90% > J3				46% ± 0.5DC		
Intracor	189	6M-18M	20/24	J3	J2	9% < −2lns	11% < −2lns	---	---	---
			77% > 20/25	66% > J2						
KAMRA	166	6M-4Y	20/17	J3	---	5% < −2lns	5% < −2lns	---	1%	6%
			93% > 20/25	73% > J2						
**PresbyMAX**	**892**	**6M-1Y**	**20/24**	**J1**	**J3**	**5% < −2lns**	**3% < −2lns**	**85% ± 0.5DS**	**10%**	**1%**
			**77% > 20/25**	**90% > J2**	**38% > +2lns**			**98% ± 0.5DC**		

UDVA: uncorrected distance visual acuity, UNVA: uncorrected near visual acuity, n: number of patients in the study, DCNVA: distance-corrected near visual acuity, CDVA: corrected distance visual acuity, CNVA: corrected near visual acuity, Refr. Outc.: refractive outcome, Retreat.: retreatment rate, M: months, Y: year(s), J: Jaeger scale for near vision, lns: Snellen lines, DS: dioptre sphere, DC: dioptre cylinder.

We introduced a hybrid category in the results. This category combines several methods together to benefit from their advantages and reduce the impact of their disadvantages. In other words, one can say that all corneal presbyopic correction methods have evolved to hybrid techniques that combine their original approach in different powers for either eye or the original approach with certain amount of monovision [61].

The current developments throughout the corneal presbyopic correction spectrum indicate a converging trend towards hybrid techniques.

## References

[1] Wright KW, Guemes A, Kapadia MS, Wilson SE (1999). Binocular function and patient satisfaction after monovision induced by myopic photorefractive keratectomy. J Cataract Refract Surg.

[2] Braun EH, Lee J, Steinert RF (2008). Monovision in LASIK. Ophthalmol.

[3] Vinciguerra P, Nizzola GM, Bailo G, Nizzola F, Ascari A, Epstein D (1998). Excimer laser photorefractive keratectomy for presbyopia: 24-month follow-up in three eyes. J Refract Surg.

[4] Vinciguerra P, Nizzola GM, Nizzola F, Ascari A, Azzolini M, Epstein D (1998). Zonal photorefractive keratectomy for presbyopia. J Refract Surg.

[5] Cantú R, Rosales MA, Tepichín E, Curioca A, Montes V, Bonilla J (2004). Advanced surface ablation for presbyopia using the Nidek EC-5000 laser. J Refract Surg.

[6] Alió JL, Chaubard JJ, Caliz A, Sala E, Patel S (2006). Correction of presbyopia by technovision central multifocal LASIK (presbyLASIK). J Refract Surg.

[7] Charman WN (2004). Ablation design in relation to spatial frequency, depth-of-focus, and age. J Refract Surg.

[8] Artola A, Patel S, Schimchak P, Ayala MJ, Ruiz-Moreno JM, Alió JL (2006). Evidence for delayed presbyopia after photorefractive keratectomy for myopia. Ophthalmology.

[9] Dai GM (2006). Optical surface optimization for the correction of presbyopia. Appl Opt.

[10] Ortiz D, Alió JL, Illueca C, Mas D, Sala E, Pérez J, Espinosa J (2007). Optical analysis of presbyLASIK treatment by a light propagation algorithm. J Refract Surg.

[11] Becker KA, Jaksche A, Holz FG (2006). PresbyLASIK: treatment approaches with the excimer laser. Ophthalmologe.

[12] Telandro A (2004). Pseudo-accommodative cornea: a new concept for correction of presbyopia. J Refract Surg.

[13] Luger MHA, Ewering T, Arba Mosquera S (2013). 1-year experience in presbyopia correction with bi-aspheric multifocal central presbyLASIK. Cornea.

[14] Baudu P, Penin F, Arba Mosquera S (2013). Uncorrected binocular performance after bi-aspheric ablation profile for presbyopic corneal treatment using AMARIS with PresbyMAX module. Am J Ophthalmol.

[15] Reinstein DZ, Couch DG, Archer TJ (2009). LASIK for hyperopic astigmatism and presbyopia using micro-monovision with the Carl Zeiss Meditec MEL80 platform. J Refract Surg.

[16] Epstein RL, Gurgos MA (2009). Presbyopia treatment by monocular peripheral presbyLASIK. J Refract Surg.

[17] Ayoubi MG, Leccisotti A, Goodall EA, McGilligan VE, Moore TC (2010). Femtosecond laser in situ keratomileusis versus conductive keratoplasty to obtain monovision in patients with emmetropic presbyopia. J Cataract Refract Surg.

[18] Reilly CD, Lee WB, Alvarenga L, Caspar J, Garcia-Ferrer F, Mannis MJ (2006). Surgical monovision and monovision reversal in LASIK. Cornea.

[19] Goldberg DB (2001). Laser in situ keratomileusis monovision. J Cataract Refract Surg.

[20] Goldberg DB (2003). Comparison of myopes and hyperopes after laser in situ keratomileusis monovision. J Cataract Refract Surg.

[21] Garcia-Gonzalez M, Teus MA, Hernandez-Verdejo JL (2010). Visual outcomes of LASIK-induced monovision in myopic patients with presbyopia. Am J Ophthalmol.

[22] Ito M, Shimizu K, Iida Y, Amano R (2012). Five-year clinical study of patients with pseudophakic monovision. J Cataract Refract Surg.

[23] Stahl JE (2007). Conductive keratoplasty for presbyopia: 3-year results. J Refract Surg.

[24] Ye P, Xu W, Tang X, Yao K, Li Z, Xu H, Shi J (2011). Conductive keratoplasty for symptomatic presbyopia following monofocal intraocular lens implantation. Clin Exp Ophthalmol.

[25] Jackson WB, Tuan KM, Mintsioulis G (2011). Aspheric wavefront-guided LASIK to treat hyperopic presbyopia: 12-month results with the VISX platform. J Refract Surg.

[26] Pinelli R, Ortiz D, Simonetto A, Bacchi C, Sala E, Alió JL (2008). Correction of presbyopia in hyperopia with a center-distance, paracentral-near technique using the Technolas 217z platform. J Refract Surg.

[27] Uy E, Go R (2009). Pseudoaccommodative cornea treatment using the NIDEK EC-5000 CXIII excimer laser in myopic and hyperopic presbyopes. J Refract Surg.

[28] Jung SW, Kim MJ, Park SH, Joo CK (2008). Multifocal corneal ablation for hyperopic presbyopes. J Refract Surg.

[29] Ryan A, O’Keefe M (2013). Corneal approach to hyperopic presbyopia treatment: six-month outcomes of a new multifocal excimer laser in situ keratomileusis procedure. J Cataract Refract Surg.

[30] Cosar CB, Sener AB (2014). Supracor hyperopia and presbyopia correction: 6-month results. Eur J Ophthalmol.

[31] Uthoff D, Pölzl M, Hepper D, Holland D (2012). A new method of cornea modulation with excimer laser for simultaneous correction of presbyopia and ametropia. Graefes Arch Clin Exp Ophthalmol.

[32] Bohac M, Gabrić N, Anticić M, Draca N, Dekaris I (2011). First results of Intracor procedure in Croatia. Coll Antropol.

[33] Holzer MP, Mannsfeld A, Ehmer A, Auffarth GU (2009). Early outcomes of INTRACOR femtosecond laser treatment for presbyopia. J Refract Surg.

[34] Ruiz LA, Cepeda LM, Fuentes VC (2009). Intrastromal correction of presbyopia using a femtosecond laser system. J Refract Surg.

[35] Holzer MP, Knorz MC, Tomalla M, Neuhann TM, Auffarth GU (2012). Intrastromal femtosecond laser presbyopia correction: 1-year results of a multicenter study. J Refract Surg.

[36] Menassa N, Fitting A, Auffarth GU, Holzer MP (2012). Visual outcomes and corneal changes after intrastromal femtosecond laser correction of presbyopia. J Cataract Refract Surg.

[37] Lindstrom RL, Macrae SM, Pepose JS, Hoopes PC (2013). Corneal inlays for presbyopia correction. Curr Opin Ophthalmol.

[38] Tomita M, Kanamori T, Waring GO, Nakamura T, Yukawa S (2013). Small-aperture corneal inlay implantation to treat presbyopia after laser in situ keratomileusis. J Cataract Refract Surg.

[39] Yılmaz OF, Alagöz N, Pekel G, Azman E, Aksoy EF, Cakır H, Bozkurt E, Demirok A (2011). Intracorneal inlay to correct presbyopia: Long-term results. J Cataract Refract Surg.

[40] Seyeddain O, Riha W, Hohensinn M, Nix G, Dexl AK, Grabner G (2010). Refractive surgical correction of presbyopia with the AcuFocus small aperture corneal inlay: two-year follow-up. J Refract Surg.

[41] Dexl AK, Seyeddain O, Riha W, Hohensinn M, Hitzl W, Grabner G (2011). Reading performance after implantation of a small-aperture corneal inlay for the surgical correction of presbyopia: Two-year follow-up. J Cataract Refract Surg.

[42] Dexl AK, Seyeddain O, Riha W, Hohensinn M, Rückl T, Reischl V, Grabner G (2012). One-year visual outcomes and patient satisfaction after surgical correction of presbyopia with an intracorneal inlay of a new design. J Cataract Refract Surg.

[43] Tomita M, Kanamori T, Waring GO, Yukawa S, Yamamoto T, Sekiya K, Tsuru T (2012). Simultaneous corneal inlay implantation and laser in situ keratomileusis for presbyopia in patients with hyperopia, myopia, or emmetropia: six-month results. J Cataract Refract Surg.

[44] Dexl AK, Seyeddain O, Riha W, Rückl T, Bachernegg A, Emesz M, Ruckhofer J, Grabner G (2012). Reading performance and patient satisfaction after corneal inlay implantation for presbyopia correction: two-year follow-up. J Cataract Refract Surg.

[45] Alarcón A, Anera RG, Villa C, Jiménez Del Barco L, Gutierrez R (2011). Visual quality after monovision correction by laser in situ keratomileusis in presbyopic patients. J Cataract Refract Surg.

[46] Reinstein DZ, Archer TJ, Gobbe M (2011). Aspheric ablation profile for presbyopic corneal treatment using the MEL80 and CRS Master Laser Blended Vision module. J Emmetropia.

[47] Johannsdottir KR, Stelmach LB (2001). Monovision: a review of the scientific literature. Optom Vis Sci.

[48] Patel S, Alió JL, Feinbaum C (2008). Comparison of Acri.Smart multifocal IOL, crystalens AT-45 accommodative IOL, and Technovision presbyLASIK for correcting presbyopia. J Refract Surg.

[49] Miranda D, Krueger RR (2004). Monovision laser in situ keratomileusis for pre-presbyopic and presbyopic patients. J Refract Surg.

[50] Hersh PS (2005). Optics of conductive keratoplasty: implications for presbyopia management. Trans Am Ophthalmol Soc.

[51] Cheng AC, Lam DS (2005). Monovision LASIK for pre-presbyopic and presbyopic patients. J Refract Surg.

[52] Jain S, Ou R, Azar DT (2001). Monovision outcomes in presbyopic individuals after refractive surgery. Ophthalmol.

[53] Illueca C, Alió JL, Mas D, Ortiz D, Pérez J, Espinosa J, Esperanza S (2008). Pseudoaccommodation and visual acuity with Technovision presbyLASIK and a theoretical simulated Array multifocal intraocular lens. J Refract Surg.

[54] Alió JL, Amparo F, Ortiz D, Moreno L (2009). Corneal multifocality with excimer laser for presbyopia correction. Curr Opin Ophthalmol.

[55] Semoun O, Bourcier T, Dupas B, Puech M, Maftouhi AE, Borderie V, Laroche L (2008). Early bacterial keratitis after presbyopic LASIK. Cornea.

[56] Luger MH, Ewering T, Arba-Mosquera S (2014). Nonwavefront-guided presby reversal treatment targeting a monofocal cornea after Bi-aspheric ablation profile in a patient intolerant to multifocality. J Refract Surg.

[57] Taneri S, Oehler S (2013). Keratectasia after treating presbyopia with INTRACOR followed by SUPRACOR enhancement. J Refract Surg.

[58] Tabernero J, Artal P (2012). Optical modeling of a corneal inlay in real eyes to increase depth of focus: optimum centration and residual defocus. J Cataract Refract Surg.

[59] Tabernero J, Schwarz C, Fernández EJ, Artal P (2011). Binocular visual simulation of a corneal inlay to increase depth of focus. Invest Ophthalmol Vis Sci.

[60] Langenbucher A, Goebels S, Szentmáry N, Seitz B, Eppig T (2013). Vignetting and field of view with the KAMRA corneal inlay. Biomed Res Int.

[61] Zheleznyak L, Sabesan R, Oh JS, MacRae S, Yoon G (2013). Modified monovision with spherical aberration to improve presbyopic through-focus visual performance. Invest Ophthalmol Vis Sci.

